# Supplementary material for: Retinotopic-like maps of spatial sound in primary ‘visual’ cortex of blind human echolocators
Source: Proc Biol Sci. 2019 Oct 2;286(1912):20191910. doi: 10.1098/rspb.2019.1910 (PMC6790759; doi:10.1098/rspb.2019.1910)
Supplement: Supplementary information on methods and results [file rspb20191910supp1.pdf]

Supplementary Information

Retinotopic-like maps of spatial sound in primary 'visual' cortex of blind human echolocators

Authors: Liam J. Norman[1] and Lore Thaler[1*]
[1]*Department of Psychology, Durham University, Durham, DH1 3LE*
**Corresponding author, lore.thaler@durham.ac.uk, tel:* +44 (0)191 3343290

Contents

Sound stimuli and recording/playback equipment 2
MRI scanning procedures 3
Retinotopic mapping of visual stimuli in sighted participants 4
Images of phase encoded maps in primary 'visual' cortex of sighted control participants 5
Summary table of retinotopic-like mapping values in primary 'visual' cortex 8
Summary table of chance level of retinotopic-like mapping values in primary 'visual' cortex 9
Single case statistics to test whether expert echolocators have greater retinotopic-like mapping compared to controls 10
Analysis of retinotopic-like mapping of echoes in EE1, EE3 and EE5, separated by laterality and eccentricity 10
Analysis of retinotopic-like mapping of source sounds in EE5, separated by laterality and eccentricity 11
Summary table of retinotopic-like mapping values in V2 and V3 12
Analysis of contralateral mapping in primary visual cortex 14
  Contralateral mapping of echo sounds - results 15
  Contralateral mapping of source sounds - results 16
Psychophysical measurement of echo and source sound localisation ability (post-MRI) 18
Participant details 21
Acoustic analysis of sound stimuli 22
References 23

Sound stimuli and recording/playback equipment

Recordings were made at a sampling rate of 96 kHz and resolution of 24-bit using a portable digital recorder (Tascam DR-100 MK2, TEAC Corporation, Japan) and in-ear microphones (Bruel & Kjaer model 4101, Denmark). Recordings were made in a 2.9 m × 4.2 m × 4.9 m noise insulated and echo dampened room (walls and ceiling lined with foam wedges with cut-off frequency 315 Hz; floor covered with foam baffles, noise floor 24 dBA). During sound recording, the participant stood in the centre of the room and rested their chin on top of a pole, adjusted to their height, to maintain a constant head level. Tactile cues were used in order to allow participants to maintain a consistent position of their head facing the centre of the rear wall. Head position was also monitored visually by the experimenters. For recording of the echo sounds, a loudspeaker (Fostex FE103En) mounted on a metal pole (1 cm diameter) was positioned in front of the participant's mouth, facing directly away from the participant. The loudspeaker was driven by a Dell Latitude E7470 laptop (Intel Core i56300U CPU 2.40 GHz, 8 GB RAM, 64-bit Windows 7 Enterprise) through a USB Soundcard (Creative Sound Blaster X-Fi HD Sound Card; Creative Technology Ltd., Creative Labs Ireland, Dublin, Ireland) and amplified by a Kramer 900N Stereo Power Amplifier (Kramer Electronics Ltd., Jerusalem, Israel). The loudspeaker was used to emit an artificial click lasting ~3 ms, modelled after real human mouth clicks (Thaler et al., 2018) and created using MATLAB (R2015b, The Mathworks, Natick, MA) at a sampling rate of 96 kHz and resolution of 24-bit.

Recorded sound files were filtered to achieve frequency response equalisation for playback through the MRI-compatible insert earphones (model S-14, Sensimetrics, Malden, MA; filters provided by the manufacturer). These earphones were amplified by a Kramer 900N Stereo Power Amplifier (Kramer Electronics Ltd., Jerusalem, Israel), with input provided by a USB Soundcard (Creative Sound Blaster X-Fi HD Sound Card; Creative Technology Ltd., Creative Labs Ireland, Dublin, Ireland). Sounds were played to participants at a level at which the highest peak intensity was presented at 80 dB SPL, and

the same sound level was used for echo and source-sound conditions, and also for psychophysical tests outside the scanner.

MRI scanning procedures

Imaging was performed at Durham University Neuroimaging Facility (James Cook University Hospital, Middlesbrough, UK), with a 3-Tesla, whole-body MRI system (Magnetom Tim Trio; Siemens, Erlangen, Germany) and 32-channel head coil. A T1-weighted, optimised sequence (MP RAGE) was used to acquire high-resolution structural images for each participant, at a resolution of 1 x 1 x 1 mm. A single-shot gradient echo-planar pulse sequence in combination with a sparse sampling design [23] was used to acquire functional images, with a repetition time of 10 seconds (2 s slice acquisition + 8 s silent gap). Field of view was 240 mm with a matrix size of 80 x 80, giving an in-slice resolution of 3 mm. 36 contiguous axial slices were acquired in ascending order with a slice thickness of 3 mm, covering the whole brain. Echo time was 30 ms and flip angle was 78°.

Participants wore MRI compatible insert earphones (model S-14, Sensimetrics, Malden, MA) for all functional runs, and these were encased in replaceable foam tips that provided a 20 to 40 dB attenuation level (information provided by the manufacturer). To minimize background noise, the MRI bore’s circulatory air fan was turned off during experimental runs. To minimise interference from light sources, all lights inside the MRI room were turned off and, for the echo and source sound conditions, participants who were not totally blind wore a blindfold.

Retinotopic mapping of visual stimuli in sighted participants

A custom-built apparatus placed inside the bore of the scanner was used in order to present visual stimuli at the same eccentricity coordinates as those used for the auditory stimuli. The apparatus was a curved plastic panel positioned in between the head coil and the internal wall of the scanner bore (see Figure 4c). Once the participant was comfortable inside the scanner, the stimulus panel was positioned in depth to be level with the participant's eyes. At 8 points along the inside of the panel (L40°, L20°, L10°, L05°, R05°, R10°, R20°, and R40°) as well as the central location (0°), end-emitting fibre optic filaments were used to provide light stimuli to participants as they lay inside of the MRI scanner. Red LEDs were used as light sources for the stimulus positions and a white LED for the central location, which acted as a fixation point for participants. The diameters of the filament ends were 3 mm. The white fixation LED remained on throughout the experiment, and during each trial one of the red stimulus LEDs would flicker on/off at a rate of 1.25 Hz for a period of 8 s. The LED display was driven via the digital I/O port on a Cambridge Research Systems ViSaGe MKII Stimulus Generator using a custom MATLAB script. During the experiment all other light sources in the room were turned off. Before beginning the experiment, a check was run to ensure that the participants could see all 8 red stimulus lights whilst fixating the white light.

To estimate a practical upper limit for the degree of retinotopic-like mapping of sound echoes (or sound sources) that we might expect in our data (i.e. considering limitations of sparse sampling fMRI design and the accuracy of a probabilistic retinotopic map for eccentricity based on cortical anatomy) we determined correlations between the observed visual stimulus map for eccentricity (in sighted participants) and the predicted map based on the probabilistic atlas. Overall these correlations were moderate to strong (mean r = 0.59, SD = 0.13). This upper limit is indicated by the horizontal cyan lines in Figure 3 in the main article

Images of phase encoded maps in primary 'visual' cortex of sighted control participants

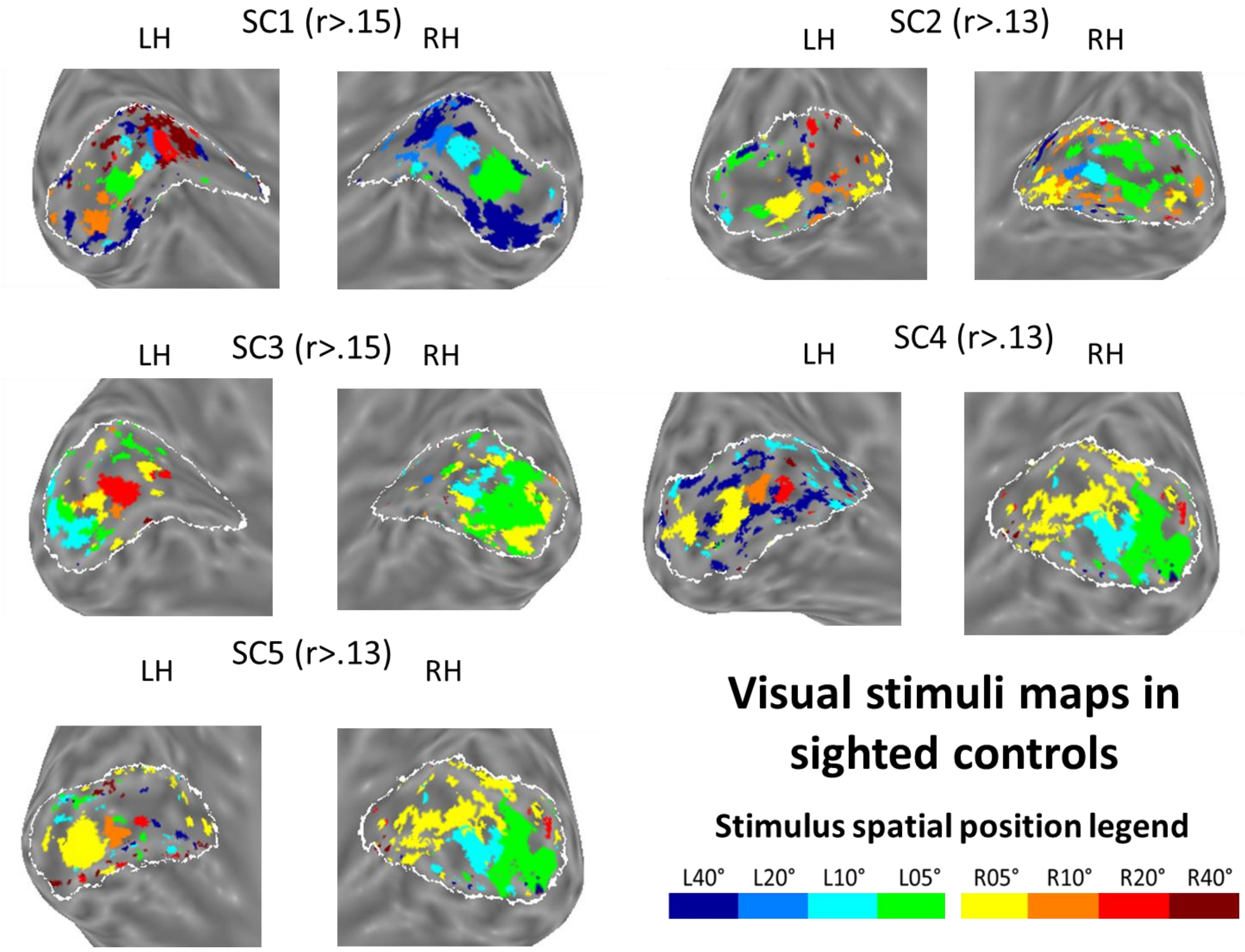

Supplementary Figure S1 Phase-encoded stimulus maps for visual stimuli in primary 'visual' cortex of sighted controls. Each pair of images shows inflated cortical surface views of left and right primary 'visual' cortex for each participant (SC1 to SC5). Each voxel in primary 'visual' cortex is color-coded to indicate the stimulus position with which the voxel's activity was correlated most strongly. The color-coded legend for spatial position is shown in the bottom right. To aid with visualization, only voxels above a correlation coherence threshold are color-coded (this value is shown beside each participant identifier). The coherence threshold is set individually for each participant but is fixed across stimulus conditions (echo/source/vision). All voxels in primary 'visual' cortex were included in the statistical analyses reported in the results section. The white outline on each image shows the boundary of primary 'visual' cortex as defined by the probabilistic atlas used (Benson *et al*, 2014).

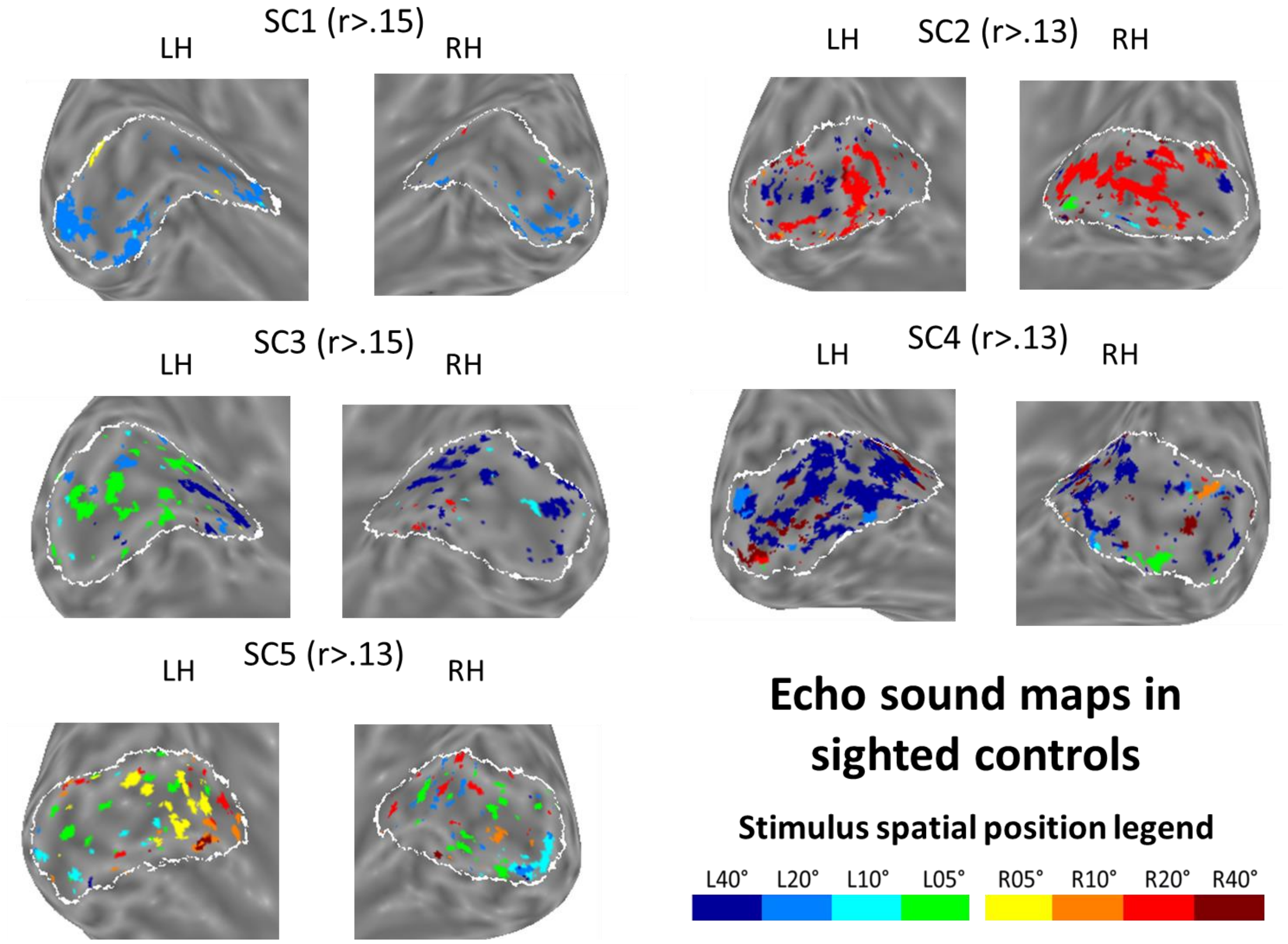

Supplementary Figure S2 Phase-encoded stimulus maps for echo sounds in primary 'visual' cortex of sighted controls. Each pair of images shows inflated cortical surface views of left and right primary 'visual' cortex for each participant (SC1 to SC5). Each voxel in primary 'visual' cortex is color-coded to indicate the stimulus position with which the voxel's activity was correlated most strongly. The color-coded legend for spatial position is shown in the bottom right. To aid with visualization, only voxels above a correlation coherence threshold are color-coded (this value is shown beside each participant identifier). The coherence threshold is set individually for each participant but is fixed across stimulus conditions (echo/source/vision). All voxels in primary 'visual' cortex were included in the statistical analyses reported in the results section. The white outline on each image shows the boundary of primary 'visual' cortex as defined by the probabilistic atlas used (Benson *et al*, 2014).

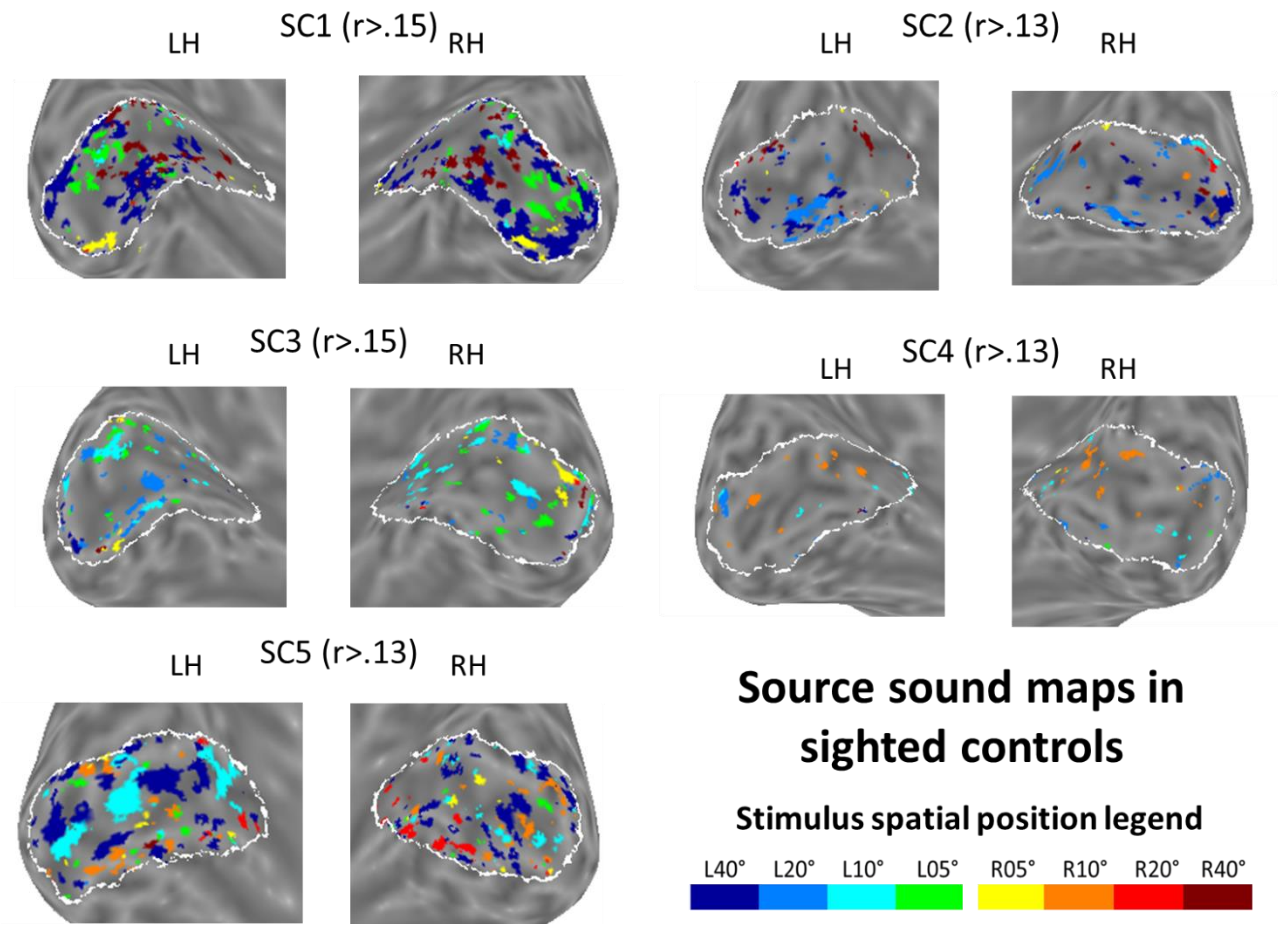

Supplementary Figure S3 Phase-encoded stimulus maps for source sounds in primary 'visual' cortex of sighted controls. Each pair of images shows inflated cortical surface views of left and right primary 'visual' cortex for each participant (SC1 to SC5). Each voxel in primary 'visual' cortex is color-coded to indicate the stimulus position with which the voxel's activity was correlated most strongly. The color-coded legend for spatial position is shown in the bottom right. To aid with visualization, only voxels above a correlation coherence threshold are color-coded (this value is shown beside each participant identifier). The coherence threshold is set individually for each participant but is fixed across stimulus conditions (echo/source/vision). All voxels in primary 'visual' cortex were included in the statistical analyses reported in the results section. The white outline on each image shows the boundary of primary 'visual' cortex as defined by the probabilistic atlas used (Benson *et al*, 2014).

Summary table of retinotopic-like mapping values in primary 'visual' cortex

Supplementary Table S1 Results summary table showing the degree of retinotopic-like mapping of echo sounds, source sounds and visual stimuli in V1 (columns 2, 3 and 4, respectively). Each score represents the correlation between the observed stimulus map and one predicted based on cortical anatomy. In each case, 95% confidence limits for each score are shown in parentheses. Confidence limits were acquired using a bootstrapping procedure (using matlab's *bootstrp* function) to calculate correlation coefficients on 1000 resampled variations of the data. EE refers to echolocation experts, BC to blind controls, and SC to sighted controls.

Participant ID	Mapping of echo sounds	Mapping of source sounds	Mapping of visual stimuli
EE 1	0.25 (0.23, 0.27)	0.17 (0.14, 0.19)	n/a
EE 2	0.02 (0.01, 0.04)	-0.03 (-0.05, -0.01)	n/a
EE 3	0.37 (0.36, 0.39)	0.17 (0.16, 0.19)	n/a
EE 4	0.06 (0.04, 0.07)	-0.05 (-0.07, -0.03)	n/a
EE 5	0.19 (0.16, 0.21)	0.26 (0.24, 0.28)	n/a
**EE mean**	**0.18**	**0.10**	**n/a**
BC 1	-0.01 (-0.03, 0.01)	-0.03 (-0.05, -0.01)	n/a
BC 2	0.08 (0.06, 0.10)	0.10 (0.08, 0.12)	n/a
BC 3	0.10 (0.08, 0.12)	0.06 (0.04, 0.09)	n/a
BC 4	-0.04 (-0.05, -0.02)	0.02 (0.01, 0.04)	n/a
BC 5	0.00 (-0.02, 0.02)	0.09 (0.07, 0.10)	n/a
**BC mean**	**0.03**	**0.05**	**n/a**
SC 1	-0.05 (-0.07, -0.04)	0.18 (0.16, 0.20)	0.54 (0.52, 0.55)
SC 2	-0.13 (-0.15, -0.11)	0.07 (0.05, 0.09)	0.67 (0.65, 0.70)
SC 3	0.09 (0.07, 0.11)	0.00 (-0.01, 0.02)	0.74 (0.72, 0.76)
SC 4	0.05 (0.03, 0.07)	0.02 (0.00, 0.03)	0.40 (0.37, 0.43)
SC 5	-0.02 (-0.04, 0.00)	-0.04 (-0.06, -0.02)	0.59 (0.55, 0.63)
**SC mean**	**-0.01**	**0.05**	**0.59**
**Overall mean**	**0.06**	**0.07**	**0.59**

Summary table of chance level of retinotopic-like mapping values in primary 'visual' cortex

Supplementary Table S2 Results summary table showing the correlation expected by chance between observed neural maps and those predicted based on cortical anatomy in V1. The values of the observed neural map were randomly shuffled and then correlated with those predicted based on cortical anatomy. This was done 1000 times for each map and the average correlation coefficient was taken (shown below). In each case, 95% confidence limits for each score are shown in parentheses, based on the standard error of the coefficients from the randomised data. EE refers to echolocation experts, BC to blind controls, and SC to sighted controls.

Participant ID	Mapping of echo sounds	Mapping of source sounds	Mapping of visual stimuli
EE 1	0.00 (-0.02, 0.02)	0.00 (-0.02, 0.02)	n/a
EE 2	0.00 (-0.02, 0.02)	0.00 (-0.02, 0.02)	n/a
EE 3	0.00 (-0.02, 0.02)	0.00 (-0.02, 0.02)	n/a
EE 4	0.00 (-0.02, 0.02)	0.00 (-0.02, 0.02)	n/a
EE 5	0.00 (-0.02, 0.02)	0.00 (-0.02, 0.02)	n/a
**EE mean**	**0.00**	**0.00**	**n/a**
BC 1	0.00 (-0.02, 0.02)	0.00 (-0.02, 0.02)	n/a
BC 2	0.00 (-0.02, 0.02)	0.00 (-0.02, 0.02)	n/a
BC 3	0.00 (-0.02, 0.02)	0.00 (-0.02, 0.02)	n/a
BC 4	0.00 (-0.02, 0.02)	0.00 (-0.02, 0.02)	n/a
BC 5	0.00 (-0.02, 0.02)	0.00 (-0.02, 0.02)	n/a
**BC mean**	**0.00**	**0.00**	**n/a**
SC 1	0.00 (-0.02, 0.02)	0.00 (-0.02, 0.02)	0.00 (-0.02, 0.02)
SC 2	0.00 (-0.02, 0.02)	0.00 (-0.02, 0.02)	0.00 (-0.03, 0.03
SC 3	0.00 (-0.02, 0.02)	0.00 (-0.02, 0.02)	0.00 (-0.02, 0.02)
SC 4	0.00 (-0.02, 0.02)	0.00 (-0.02, 0.02)	0.00 (-0.03, 0.03)
SC 5	0.00 (-0.02, 0.02)	0.00 (-0.02, 0.02)	0.00 (-0.03, 0.03)
**SC mean**	**0.00**	**0.00**	**0.00**
**Overall mean**	**0.00**	**0.00**	**0.00**

Single case statistics to test whether expert echolocators have greater retinotopic-like mapping compared to controls

In addition to the group statistics reported in the main document, we also performed a separate statistical test for each expert echolocator (EE) to determine whether they had a significantly higher degree of retinotopic-like mapping of echo sounds compared to the ten control participants (sighted and blind controls combined). Each of these tests was a modified t-tests (Crawford & Howell, 1998; Crawford & Garthwaite, 2002), which tests whether a single case differs significantly from a control group. Compared to the control participants (mean=0.007, SD=0.073), three EEs had significantly higher retinotopic-like mapping (EE1=0.246, $t_{(9)}$=3.143, p=0.012, 95% CI=[0.067, 0.411][1]; EE3=0.375, $t_{(9)}$=4.836, p<0.001, 95% CI=[0.196, 0.540]; EE5=0.187, $t_{(9)}$=2.364, p=0.042, 95% CI=[0.008 0.352]) but not two of them (EE2=0.023, $t_{(9)}$=0.210, p=0.839, 95% CI=[-0.156 0.188]; EE4=0.058, $t_{(9)}$=0.669, p=0.520, 95% CI=[-0.121, 0.223]).

For source sounds, compared to the control participants (mean=0.048, SD=0.067), one EE had significantly higher retinotopic-like mapping (EE5=0.259, $t_{(9)}$=3.025, p=0.014, 95% CI=[0.053 0.369]). The remaining four did not (EE1=0.167, $t_{(9)}$=1.708, p=0.122, 95% CI=[-0.039 0.277]; EE2=-0.034, $t_{(9)}$=1.167, p=0.273, 95% CI=[-0.239 0.076]; EE3=0.173, $t_{(9)}$=1.794, p=0.106, 95% CI=[-0.033 0.283]; EE4=-0.051, $t_{(9)}$=1.405, p=0.194, 95% CI=[-0.256 0.060]).

Analysis of retinotopic-like mapping of echoes in EE1, EE3 and EE5, separated by laterality and eccentricity

Our main analysis suggest that, in EEs 1, 3 and 5, there is retinotopic-like mapping of echo sounds in primary visual cortex. To what degree is this effect driven by mapping of laterality or eccentricity? In order to test this, we performed further analyses on the mapping data in these three EEs. We ran

[1] These confidence intervals refer to the *difference* between the EE's score and the group score.

two separate linear regression models on the mapping data for each participant – one that used the retinotopic atlas as a predictor only of laterality (i.e. all values in the atlas and in the observed maps were converted to either -1 or 1 to represent left and right space, respectively), and one that used the atlas as a predictor only of eccentricity (i.e. the absolute values of all values in the atlas and in the observed maps were taken to represent the distance from central space). In EE1 the effect of laterality was significant (beta = 0.427, $t_{(8140)}$ = 42.900, p<0.001), but the effect of eccentricity was not significant (beta = -0.070, $t_{(8140)}$ = 0.765, p=0.444). In EE3 the effect of laterality was significant (beta = 0.425, $t_{(9895)}$ = 46.891, p<0.001) and the effect of eccentricity was also significant (beta = 0.052, $t_{(8140)}$ = 6.663, p<0.001). In EE5 the effect of laterality was significant (beta = 0.213, $t_{(9076)}$ = 20.726, p<0.001) and the effect of eccentricity was also significant (beta = 0.205, $t_{(9076)}$ = 27.221, p<0.001). These results suggest that in two EEs (EE3 and EE5), the retinotopic-like mapping of echo sounds can explained by mapping of both laterality and eccentricity.

Analysis of retinotopic-like mapping of source sounds in EE5, separated by laterality and eccentricity

As with the previous analysis described for the retinotopic-like mapping of echo sounds in EEs 1, 3 and 5, we tested whether the retinotopic-like mapping of source sounds in EE5 (the only EE to show significantly greater retinotopic-like mapping compared to controls) is driven by mapping of both contralaterality and eccentricity. In EE5 the effect of laterality was significant (beta = 0.217, $t_{(9076)}$ = 21.285, p<0.001) and the effect of eccentricity was also significant (beta = 0.03, $t_{(9076)}$ = 3.704, p<0.001). These results suggest that in one EE (EE5), the retinotopic-like mapping of echo sounds can explained by mapping of both laterality and eccentricity.

Summary table of retinotopic-like mapping values in V2 and V3

Whilst for theoretical reason the main focus of our manuscript is on V1, we also ran analysis for V2 and V3. Both V2 and V3 each contain a complete retinotopically organised map of visual space (Wandell & Winawer, 2011), and there is evidence of direct connectivity between auditory cortex and these early visual areas (Beer A, Plank T, & Greenlee M, 2011; Cate et al, 2009; Ungerleider & Desimone, 1986).Thus, we carried out further analyses in order map the neural responses to echo and source sounds in visual areas V2 and V3 (the second and third visual cortical areas). Specifically, the probabilistic atlas that we used in our main analysis provides an expected retinotopic map also for V2 and V3, so we ran the same analysis to quantify retinotopic-like mapping of echo and source sounds (and for visual stimuli in sighted participants) in V2 and V3 as we did for V1. These data are shown in supplementary tables S3 and S4. It is evident that for those EEs where we found retinotopic-like mapping of echo and source sounds in V1, we also find it in V2 and V3, suggesting that retinotopic-like mapping of echo and source sounds extends to cortex beyond the primary visual area.

In addition, to determine how reproducible (and thus reliable) maps measured in V1 are to those measured in V2 and V3 in all participants, we also correlated maps in V1 to those measured in V2 and V3. For echo sounds, the degree of retinotopic-like mapping in V1 is positively correlated with the degree of retinotopic-like mapping in V2 ($r_{(13)}$=0.779, $p<0.001$) and in V3 ($r_{(13)}$=0.830, $p<0.001$). For source sounds, the degree of retinotopic-like mapping in V1 is also positively correlated with the degree of retinotopic-like mapping in V2 ($r_{(13)}$=0.614, p=0.015) and in V3 ($r_{(13)}$=0.667, p=0.006) sounds. In sighted participants (n=5), for visual stimuli, the degree of retinotopic-like mapping in V1 is also positively correlated with the degree of retinotopic-like mapping in V2 ($r_{(3)}$=0.906, p=0.034) and (marginally significant) in V3 ($r_{(3)}$=0.829, p=0.082). The marginal significance result for V3 is due to the low sample size (n=5), as all sighted participants did show moderate-to-high retinotopic mapping of stimuli in both V2 and V3 (see Supplementary Tables S3 and S4). Overall, these results

not suggest that maps measured in V1-V3 are similar, but also that the observed neural maps in V1 are reliable and not simply the result of low statistical power.

Supplementary Table S3 Results summary table showing the degree of retinotopic-like mapping of echo sounds, source sounds and visual stimuli in V2 (columns 2, 3 and 4, respectively). Each score represents the correlation between the observed stimulus map and one predicted based on cortical anatomy. In each case, 95% confidence limits for each score are shown in parentheses. Confidence limits were acquired using a bootstrapping procedure (using matlabs *bootstrp* function) to calculate correlation coefficients on 1000 resampled variations of the data. EE refers to echolocation experts, BC to blind controls, and SC to sighted controls.

Participant ID	Mapping of echo sounds	Mapping of source sounds	Mapping of visual stimuli
EE 1	0.08 (0.06, 0.11)	0.18 (0.16, 0.20)	n/a
EE 2	-0.03 (-0.05, -0.01)	0.02 (0.00, 0.04)	n/a
EE 3	0.27 (0.25, 0.29)	0.23 (0.21, 0.25)	n/a
EE 4	0.04 (0.02, 0.05)	-0.07 (-0.08, -0.05)	n/a
EE 5	0.18 (0.15, 0.20)	0.16 (0.14, 0.18)	n/a
**EE mean**	**0.11**	**0.10**	**n/a**
BC 1	-0.01 (-0.03, 0.01)	-0.08 (-0.10, -0.06)	n/a
BC 2	0.07 (0.04, 0.09)	0.02 (0.00, 0.005)	n/a
BC 3	-0.05 (-0.08, -0.03)	0.02 (0.00, 0.005)	n/a
BC 4	0.05 (0.03,0.07)	-0.06 (-0.07, -0.04)	n/a
BC 5	-0.01 (-0.02, 0.01)	0.06 (0.04, 0.08)	n/a
**BC mean**	**0.01**	**-0.01**	**n/a**
SC 1	0.06 (0.03,0.09)	-0.11 (-0.13, -0.09)	0.54 (0.51, 0.56)
SC 2	-0.10 (-0.12, -0.08)	-0.06 (-0.08, -0.04)	0.80 (0.77, 0.83)
SC 3	0.04 (0.03, 0.06)	-0.15 (-0.16, -0.13)	0.81 (0.78, 0.84)
SC 4	-0.05 (-0.07, -0.03)	-0.05 (-0.07, -0.03)	0.23 (0.18, 0.28)
SC 5	0.03 (0.01, 0.05)	0.00 (-0.01, 0.02)	0.37 (0.28, 0.46)
**SC mean**	**0.00**	**-0.07**	**0.55**
**Overall mean**	**0.04**	**0.01**	**0.55**

Supplementary Table S4 Results summary table showing the degree of retinotopic-like mapping of echo sounds, source sounds and visual stimuli in V3 (columns 2, 3 and 4, respectively). Each score represents the correlation between the observed stimulus map and one predicted based on cortical anatomy. In each case, 95% confidence limits for each score are shown in parentheses. Confidence limits were acquired using a bootstrapping procedure (using matlabs *bootstrp* function) to calculate correlation coefficients on 1000 resampled variations of the data. EE refers to echolocation experts, BC to blind controls, and SC to sighted controls.

Participant ID	Mapping of echo sounds	Mapping of source sounds	Mapping of visual stimuli
EE 1	0.15 (0.13, 0.17)	0.15 (0.13, 0.18)	n/a
EE 2	0.16 (0.14, 0.18)	0.03 (0.01, 0.05)	n/a
EE 3	0.26 (0.24, 0.28)	0.11 (0.10, 0.13)	n/a
EE 4	-0.03 (-0.05, -0.01)	-0.05 (-0.07, -0.03)	n/a
EE 5	0.24 (0.22, 0.26)	0.21 (0.19, 0.23)	n/a
**EE mean**	**0.16**	**0.09**	**n/a**
BC 1	-0.05 (-0.06, -0.03)	-0.02 (-0.04, -0.01)	n/a
BC 2	0.10 (0.08, 0.12)	0.05 (0.03, 0.08)	n/a
BC 3	0.07 (0.05, 0.09)	0.12 (0.10, 0.14)	n/a
BC 4	-0.01 (-0.03, 0.00)	-0.10 (-0.12, -0.08)	n/a
BC 5	0.06 (0.05, 0.08)	-0.06 (-0.08, -0.05)	n/a
**BC mean**	**0.04**	**0.00**	**n/a**
SC 1	0.01 (0.00, 0.03)	0.00 (-0.02, 0.02)	0.60 (0.58, 0.62)
SC 2	-0.05 (-0.07, -0.03)	0.04 (0.03, 0.06)	0.70 (0.66, 0.73)
SC 3	0.06 (0.04, 0.08)	-0.11 (-0.13, -0.09)	0.73 (0.70, 0.76)
SC 4	0.06 (0.04, 0.08)	-0.05 (-0.07, -0.03)	0.57 (0.53, 0.62)
SC 5	0.04 (0.02, 0.06)	0.03 (0.01, 0.05)	0.74 (0.71, 0.77)
**SC mean**	**0.02**	**-0.02**	**0.67**
**Overall mean**			

Analysis of contralateral mapping in primary visual cortex

The retinotopic-like mapping measure described in the main results section takes into account the full range of angular eccentricities from left to right space. In addition to this, we also measured the overall contra-lateral mapping of echo and source sounds – i.e. without taking into account eccentricity – as a coarser index of functional activity consistent with retinotopic organizational principles. A contralateral mapping index was calculated for each participant separately for the

mapping of echo and source sounds. This was calculated by first subtracting the number of voxels in left primary 'visual' cortex that mapped a left sound position from the number of voxels that mapped a right sound position, and dividing the result by the total number of mapped voxels in left primary 'visual' cortex. The same calculation was made for voxels in right primary 'visual' cortex. Then, the difference between left and right values was divided by 2 in order to give an overall contralateral mapping index. This contralateral mapping index varies from 1 (complete contralateral mapping) to -1 (complete ipsilateral mapping), with 0 indicating no overall left/right distinction.

Contralateral mapping of echo sounds - results

The contralateral mapping index is another, albeit coarser, measure to determine if the mapping of sounds in primary 'visual' cortex is consistent with retinotopic principles. Specifically, it is possible that even though control participants may not show eccentricity mapping of echo sounds, they might nonetheless show contralaterality. The contralateral mapping indices for echo sounds are shown in figure S6a. A multiple linear regression analysis was carried out using the same predictors as those used in the regression analyses reported in the main text but with the contralateral mapping index of echo sounds as the dependent variable. The regression revealed a significant effect of 'Echolocation Expertise' (standardized beta: -4.404; $t_{(10)}$=-3.359; p=.007; unstandardized B=-1.252, 95% CI=[-2.082 -0.421])[2] and a significant interaction effect between 'Echolocation Expertise' and 'Echo Localization Ability' (standardized beta: 4.835; $t_{(10)}$=3.479; p=.006; unstandardized B=1.591, 95% CI=[0.572 2.611]). None of the other predictors was significant. Following up the interaction with correlation analyses between 'Echo Localization Ability' and contralateral mapping index separately for experts and non-experts showed a positive correlation for echolocation experts ($r_{(3)}$=.916; p=.029), but no significant correlation for the other participants ($r_{(8)}$=.131; p=.718). This indicates that, for expert echolocators with high echolocation ability, primary 'visual' cortex is more likely to map the spatial locations of echoes contra-laterally but that this is not the case for the other

participants. The overall model explained 70.7% of the variance (R^2) in the contralateral mapping index, which was significant ($F_{(4,10)}$=6.024; p=.010). This suggests that contra-laterality of echo sounds is significantly associated with echolocation expertise and the interaction between expertise and echo localisation ability.

Contralateral mapping of source sounds - results

The contralateral mapping indices for source sounds are shown in figure S6b. The same multiple regression analysis applied to contralateral mapping indices for echo sounds was also applied to contralateral mapping indices for source sounds. The regression revealed a significant interaction effect between 'Echolocation expertise' and 'Echo Localization Ability' (standardized beta = 3.431; $t_{(10)}$=2.234; p=.050; unstandardized B=0.888, 95% CI=[0.002 1.775]). None of the other predictors was significant. Following up the interaction with correlation analyses between 'Echo Localization Ability' and contralateral mapping index separately for experts and non-experts showed no significant correlation for echolocation experts ($r_{(3)}$=.663; p=.223), or the other participants ($r_{(8)}$=-.288; p=.419). The overall model explained 64.2% of the variance (R^2) in the contralateral mapping index, which was significant ($F_{(4,10)}$=4.478; p=.025). This suggests that contra-laterality of source sounds is significantly associated with the interaction between expertise and echo localisation ability.

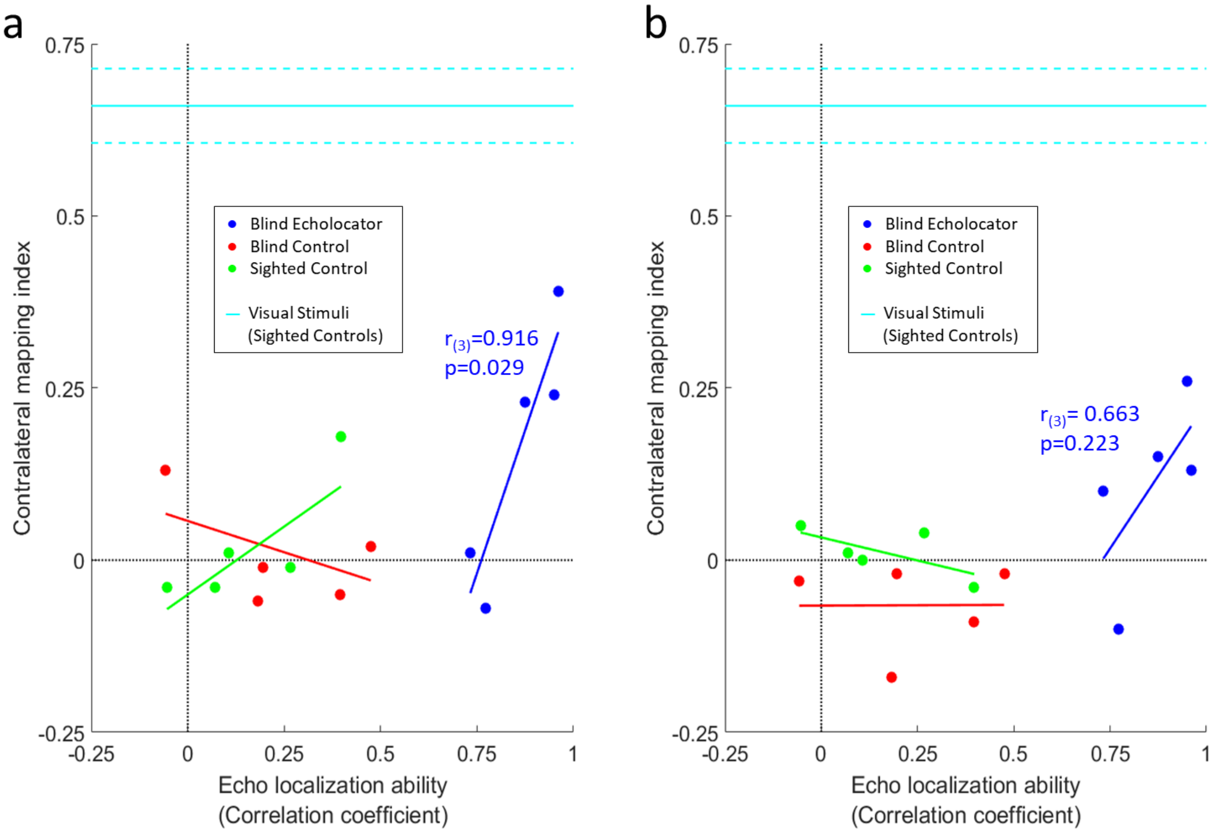

Supplementary Figure S4 (a) The association between the degree of contralateral mapping of echo sounds in calcarine cortex (y axis), echolocation expertise (separate colours) and echo localisation ability (x axis). A linear regression analysis showed that the degree of contralateral mapping of echo sounds in calcarine cortex is significantly associated with echolocation expertise and the interaction between expertise and echo localisation ability. For the expert echolocators, the degree of contralateral mapping was significantly associated with their echo localisation ability – with greater ability in localising objects through echoes, there is more contralateral mapping of echo sounds in calcarine cortex. The solid horizontal line in cyan represents the mean contralateral mapping index for visual stimuli in sighted participants (dotted lines show +/- 1 standard error of the mean). Thus, these data in cyan represent a practical upper limit for the extent of contralateral mapping of echo/source sounds, set by the limitations of a sparse sampling fMRI design. **(b)** The association between the degree of contralateral mapping of source sounds in calcarine cortex (y axis), echolocation expertise (separate colours) and echo localisation ability (x axis). Using the same predictors as those described above, the regression analysis showed that, just like for echo sounds, the degree of contralateral mapping of source sounds in primary ‘visual’ cortex calcarine cortex was significantly associated with echolocation expertise and the interaction between expertise and echo localisation ability. Yet, the overall model (and the individual correlation coefficient) was not significant, suggesting that associations are weaker as compared to those for echo sounds in our study.

Psychophysical measurement of echo and source sound localisation ability (post-MRI)

In order to quantify the ability with which each participant was able to resolve the stimulus locations used in the echo and source sound conditions, a psychophysical task was run after each participant had taken part in the fMRI component. The experiment was run with a custom MATLAB script on a Dell Latitude E7470 laptop (Intel Core i56300U CPU 2.40 GHz, 8 GB RAM, 64-bit Windows 7 Enterprise), with the same external sound equipment used in the fMRI component (soundcard, amplifier and earphones). In each trial, a stimulus from one of the 8 spatial locations was played for 8 s and followed by a tone (50 ms, 1200 Hz), at which point participants pressed one of 8 keys on a keyboard to indicate in which of the 8 possible locations they perceived the stimulus. Each block contained 48 trials presented in a random sequence (6 repetitions of each 8 stimulus location), and participants completed two blocks of trials for the echo sound task and two for the source sound task.

To measure each participant's ability for resolving the spatial locations of echo and source sound stimuli, we correlated their response values in with the actual stimulus position values to give Pearson's *r*. A higher coefficient would indicate a greater ability in resolving the stimulus locations. We chose this correlation measure to quantify psychophysical performance because it is directly analogous to the method that we use to quantify the degree of retinotopic-like mapping of stimuli in primary 'visual' cortex. A more conventional method to quantify performance, such as proportion correct, would not be as sensitive as the correlation coefficient, because proportion correct would not differentiate small errors (e.g. classifying -40 sound as -20) from large errors (e.g. classifying -40 sound as +40 sound), whereas the correlation coefficient does take this into account.). Results from this task are shown in Supplementary Table S5. As expected, EEs had better echo localisation ability

compared to controls, and all participants were very good at localising source sounds (see Supplementary Materials text and Supplementary Table S5

We performed a statistical test for each expert echolocator to determine whether they had a significantly higher degree of echo localisation ability compared to the ten control participants (sighted and blind controls combined). Each of these tests was a modified t-tests (Crawford & Howell, 1998; Crawford & Garthwaite, 2002), which tests whether a single case differs significantly from a control group. Compared to the control participants (mean=0.198, SD=0.187), all five expert echolocators (EEs) had significantly higher echo localisation ability (EE1=0.951, $t_{(9)}$=3.844, p=0.004, 95% CI=[0.310, 1.196]; EE2=0.733, $t_{(9)}$=2.730, p=0.023, 95% CI=[0.092, 0.978]; EE3=0.961, $t_{(9)}$=3.896, p=0.004, 95% CI=[0.320 1.206]; EE4=0.771, $t_{(9)}$=2.929, p=0.017, 95% CI=[0.131 1.017]; EE5=0.874, $t_{(9)}$=3.453, p=0.007, 95% CI=[0.233, 1.120]). We also did the same for source sound localisation. Compared to the control participants (mean=0.927, SD=0.038), none of the expert echolocators (EEs) had significantly higher source sound localisation ability (EE1=0.968, $t_{(9)}$=1.035, p=0.328, 95% CI=[-0.049, 0.130]; EE2=0.933, $t_{(9)}$=0.163, p=0.874, 95% CI=[-0.083, 0.096]; EE3=0.978, $t_{(9)}$=1.290, p=0.229, 95% CI=[-0.038 0.141]; EE4=0.951, $t_{(9)}$=0.623, p=0.549, 95% CI=[-0.065 0.114]; EE5=0.914, $t_{(9)}$=-0.320, p=0.756, 95% CI=[-0.102, 0.077]).

Supplementary Table S5 Results summary table showing participants' ability to localise echo and source sounds in a psychophysical task (columns 2 and 3, respectively). Each score represents the correlation (Pearson's r) between actual stimulus position and the participant's judgment of the position. In each case, 95% confidence limits for each score are shown in parentheses. Confidence limits were acquired using a bootstrapping procedure to calculate correlation coefficients on 1000 resampled variations of the data. EE refers to echolocation experts, BC to blind controls, and SC to sighted controls.

Participant ID	Echo localisation ability	Source localisation ability
EE 1	0.95 (0.94, 0.96)	0.97 (0.96, 0.98)
EE 2	0.73 (0.66, 0.81)	0.93 (0.92, 0.95)
EE 3	0.96 (0.95, 0.97)	0.98 (0.97, 0.98)
EE 4	0.77 (0.73, 0.82)	0.95 (0.94, 0.96)
EE 5	0.87 (0.84, 0.91)	0.91 (0.85, 0.98)
**EE mean**	**0.86**	**0.95**
BC 1	0.20 (0.06, 0.33)	0.85 (0.81, 0.88)
BC 2	0.40 (0.27, 0.52)	0.93 (0.91, 0.95)
BC 3	-0.06 (-0.20, 0.08)	0.96 (0.95, 0.97)
BC 4	0.48 (0.36, 0.59)	0.96 (0.95, 0.98)
BC 5	0.18 (0.04, 0.33)	0.93 (0.92, 0.95)
**BC mean**	**0.24**	**0.93**
SC 1	-0.05 (-0.21, 0.11)	0.94 (0.92, 0.95)
SC 2	0.27 (0.12, 0.41)	0.90 (0.86, 0.93)
SC 3	0.07 (-0.07, 0.21)	0.96 (0.95, 0.97)
SC 4	0.11 (-0.05, 0.26)	0.89 (0.85, 0.92)
SC 5	0.40 (0.27, 0.53)	0.95 (0.93, 0.96)
**SC mean**	**0.16**	**0.93**
**Overall mean**	**0.42**	**0.93**

Participant details

Supplementary Table S6. Details of all 15 participants, organised by participant group. EE refers to echolocation experts, BC to blind controls, and SC to sighted controls.

**Participant ID**	Age	Degree of vision loss	Cause of vision loss	Echolocation use
EE 1	50	Total blindness	Enucleation due to retinoblastoma at 13 months	Daily; since early childhood/no exact age remembered
EE 2	46	Total blindness	Enucleation at 12 months due to retinoblastoma	Daily; since 4 years old
EE 3	35	Total blindness	Gradual sight loss since birth due to glaucoma.	Daily; since 12 years old
EE 4	46	Total blindness	Enucleation at 18 months (left eye) and 30 months (right eye due to retinoblastoma	Daily; since 8-10 years old
EE 5	61	Total blindness	Optic nerve atrophy in infancy	Daily; since early childhood/no exact age remembered
BC 1	37	Residual bright light and motion perception	Hereditary retinal dystrophy; from birth	No regular use
BC 2	53	Residual bright light perception	Retinitis pigmentosa; official diagnosis age 10. Gradual sight loss from birth	Some experience; very little regular use
BC 3	61	Total blindness	Micropthalmia; from birth	No regular use
BC 4	45	Total blindness	Ocular albinism. Gradual sight loss from birth	No regular use
BC 5	45	Total blindness	Bloodclot damaging optic nerve; age 15	No regular use
SC 1	45	n/a	n/a	No regular use
SC 2	31	n/a	n/a	No regular use
SC 3	40	n/a	n/a	No regular use
SC 4	30	n/a	n/a	No regular use
SC 5	54	n/a	n/a	No regular use

Acoustic analysis of sound stimuli

Due to the nature of the stimuli, the average binaural intensity difference across stimulus positions was greater for source sounds (6.8 dB; SD 4.2) compared to echo sounds (1.3 dB; SD: 1.2). This is because, in the echo sounds the click is always central as it is emitted from the location of the mouth, and the echo is weaker compared to the source sound from the same position. We also carried out a spectral analysis of the sounds used for one of our participants (averaged across the channels). Figure S5 shows power as a function of spectral frequency for each of the different acoustic components of the sounds (i.e. source clicks, echo clicks, echoes at different positions). Compared to the clicks in the echo recordings, those in the source sound recordings contain more energy in lower frequency bands. This is to be expected given the directional characteristics of the speaker, which was positioned to face away from the participant during recording of the echo sounds, but was positioned to face towards the participant during recording of the source sounds. There are minimal changes in the spectrum of the echoes across different object positions (small differences are expected as more high frequency energy will be returned from objects that are positioned more centrally, again expected due to directional characteristics of the speaker). Importantly, however, since any acoustic differences in the stimuli apply to all participants in our study, they cannot explain the association between any neural mapping we observed and performance in the echolocation task.

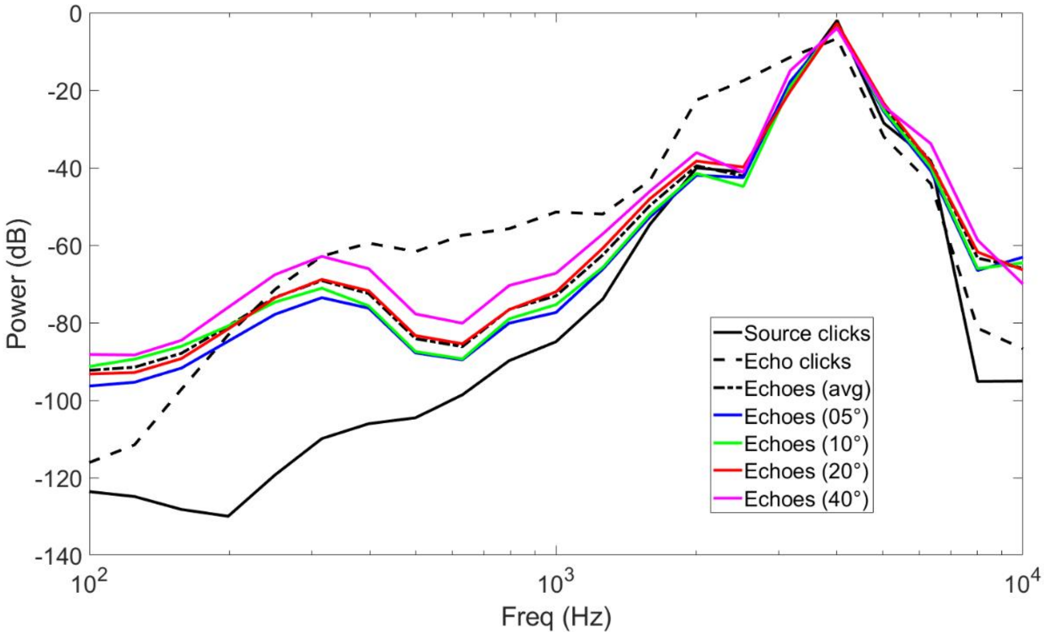

Supplementary figure S5 Power Spectra (1/3 Octave Bands with respect to total power) for each of the different acoustic components of the sounds (i.e. source clicks, echo clicks, echoes at different positions). The cut-off frequency of the MR headphones was 10kHz.

References

Beer A, Plank T, & Greenlee M. (2011). Diffusion tensor imaging shows white matter tracts between human auditory and visual cortex. *Experimental Brain Research, 213,* 299-308

Benson NC, Butt OH, Brainard DH, Aguirre GK (**2014**) Correction of distortion in flattened representations of the cortical surface allows prediction of V1-V3 functional organization from anatomy. *PLoS Comput Biol,* **10**(3):e1003538.

Cate AD, Herron, T., Yund, E., Stecker, G., Rinne, T., Kang, X., *et al.* (2009) Auditory attention activates peripheral visual cortex. *PLoS ONE 4(2),* e4645

Crawford J. R., & Howell D. C. (1998). Comparing an individual's test score against norms derived from small samples. *The Clinical Neuropsychologist*, 1998; 12(4), 482–486.

Crawford J. R., & Garthwaite P. H. (2002). Investigation of the single case in neuropsychology: Confidence limits on the abnormality of test scores and test score differences. *Neuropsychologia*, 2002; 40(8), 1196–1208.

Ungerleider, L. G. & Desimone, R. (1986) Projections to the superior temporal sulcus from the central and peripheral field representations of V1 and V2. *The Journal of Comparative Neurology 248,* 147-163

Wandell, B. A., & Winawer, J. (2011). Imaging retinotopic maps in the human brain. *Vision research*, *51*(7), 718–737. doi:10.1016/j.visres.2010.08.004
